# Holistic view of understanding genetic predisposition and perceptions of genomic research in African communities: tradition, trust and transformation

**DOI:** 10.1007/s12687-026-00889-5

**Published:** 2026-04-28

**Authors:** Nuria Ribeiro, Brendon Pearce

**Affiliations:** Genetics Department, Faculty of Agrisciences, Stellenbosch University, Van Der Bijl Street, Stellenbosch, 7600 South Africa

**Keywords:** Genomic and genetic research (GGR), Genetic Predisposition, Community engagement (CE), Genome-wide association studies (GWAS), Traditional african medicine (TAM), Ethical governance, Genomic underrepresentation, Cultural sensitivity

## Abstract

Genetic research holds immense potential to advance personalised medicine and enhance patient outcomes. However, Africa’s diverse and historically marginalised populations remain significantly underrepresented in these studies, thus limiting the relevance and reach of genomic advancements. By examining African communities through an anthropological lens, this narrative review highlights the importance of their lived realities and holistic perspectives on Genetic and Genomic Research (GGR). Beyond advocating for their inclusion, raising the importance of embedding intentional integrated medical practices, culturally sensitive ethical principles and fostering equitable long-term partnerships both locally and globally. These efforts are essential for strengthening healthcare infrastructure and ensuring that the benefits of GGR are shared fairly. Ultimately, fostering authentic participation and sustained trust in scientific research across Africa requires transparent dialogue, inclusive collaboration and a principled commitment to respecting community autonomy, not merely for the advancement of science, but to affirm the sovereignty and long-term interest of African populations.

## Introduction

The aim of this review is to examine sociocultural, historical and structural factors that influence perceptions of genetic predispositions and the integration of genomic medicine in African communities. Key themes include colonial legacies in medicine, underrepresentation in genomic research, the role of Traditional African Medicine, stigma surrounding genetic predisposition, structural and economic barriers, community engagement practices and ethical governance frameworks.

Africa is the epicentre of human genetic diversity and holds great potential for advancements in genomic and genetic research (GGR). However, it is important to recognise that the continent is not a singular entity. Africa is made up of diverse regions which are each characterised by their distinct ethnicities, cultural beliefs and social structures. These differences are shaped by the historical, socio-political and economic contexts across the Western, Eastern, Central and Southern African parts. Subsequently, such variation influences research infrastructure, community perceptions and engagement, cultural interpretations and ethical considerations surrounding GGR. While several countries have developed growing research capacity and advancements in clinical services, these resources are largely concentrated in urban centres this leaving many populations underserved. As a result, integration of these clinical advancements into healthcare systems remains unevenly accessible.

Across many African communities, historical mistrust, deep-rooted traditions of indigenous healing, social stigmas, communication barriers and structural inequities in healthcare continue to influence how science is received and interpreted. Genomics, especially when dealing with concepts like genetic predisposition, is often a personal and unfamiliar terrain (Ramsay et al. 2014). As it raises questions around identity, sovereignty, marriageability and the ethics of disclosure. These concerns demonstrate that genomic research cannot meaningfully progress under a ‘one size fits all’ model. Rather, it must be grounded in cultural sensitivity and mutual respect. Initiative such as H3Africa have played a central role in strengthening African-led genomic research, infrastructure and ethical governance. However, the translation of genomic and genetic research into routine clinical care remains limited. This reflects persistent historical inequities, resource constraints and the lived experiences that continue to shape public trust as well as of genomic research and genetic predispositions.

Whilst the rapid advancements in genomic science hold great potential (Mboowa et al. 2024), efforts towards implementation and accessibility of these revolutionary genomic tools in Africa must be approached with greater intentionality (Peprah et al. 2017). Scientific progress cannot be measured solely by publications or technological breakthroughs alone especially considering when there are communities that remain excluded and underserved from its benefits. Meaningful progress requires moving beyond the bare minimum of written consent (Tindana et al. 2017a) and to directly engage with the lived experiences, values and needs of these local populations.

These conversations must extend beyond laboratories and academic institutions, reaching into homes, schools and public spaces where real impact can unfold. Through open, sustained dialogue rooted in empathy and accountability (de Vries et al. 2016), can we foster the trust and collaborative involvement needed for long-term, community-centred participation in genomic research across nations (Staunton et al. 2018). The perspective of this review is shaped by the authors’ academic backgrounds and engagement with GGR within African contexts. The first author approaches this work with a strong interest in evolving the nature of science and its capacity not only limited to generate knowledge, but to meaningfully improve health outcomes and serve communities. This perspective informs the review’s emphasis on ethical accountability, community engagement and equitable benefit-sharing throughout the review. The second author brings expertise in genomics, pharmacogenetics and African based research ethics. Together, these positional standpoints shaped the focus on community trust, historical colonial paradigms and the need for more African-led contributions and approaches to genomic research.

## Methods

### Search strategy

This study takes on a critical narrative review approach which allows for interpretation and integrating evidence from across multiple pieces of literature that focus on the aspects of genetics, ethics, anthropology and public health particularly in the African context. In addition, an exploratory citation network analysis was performed to contextualise publication patterns and identify gaps in African-led genomic research.

To guide the search process and initial holistic map sketch of this manuscript’s topic was constructed resulting in eight key themes to be discussed as follows: (1) The significance of Africa’s genetic diversity, (2) the history of unethical research practices which have shaped mistrust within communities, (3) the relationship between Traditional African and Western medical practices, (4) the cultural and religious beliefs pertaining to genetics, (5) stigma and stereotyping within communities, (6) healthcare accessibility and infrastructure in rural areas, (7) community engagement in research and lastly, (8) initiatives that are in place that aim to promote equity and trust in genomic research.

These themes were then systematically organised and ranked in accordance with their relevance to the research question and how their inclusion would shape the overall narrative structure of the manuscript. Subsequently, each theme was further expanded into subtopics to further define key areas of inquiry. A structured table was then developed to align the themes with their corresponding subtopics.

This framework guided the identification and selection of relevant literature for the narrative review. Emphasis was placed on studies providing qualitative insights, particularly those incorporating interviews, focus groups, or first-hand accounts. Such sources were prioritised as they enriched the anthropological and psychosocial dimensions of the review therefor, offering nuanced perspectives on both individual and community-level experiences, perceptions, and attitudes towards genetic predispositions and health research.

In continuation, a systemic literature search was conducted using PubMed. Keywords and search terms such as “genomic underrepresentation”, “genetic research”, “qualitative study”, “African communities and genomic research understanding” along with Boolean operators to refine search results. Following this, Google Scholar was used to further investigate articles and external sources that took nuanced approaches upon certain subject matters discussed within the manuscript. This was particularly useful for cases where articles were published outside the required 10 year research space, thus enabling the manuscript to be reinforced with recent factual evidence. Examples of these topics include the prevalent use of traditional medicinal methods and how individuals and communities in the African context understand genetic research.

### Data extraction and analysis

**Fig. 1:**
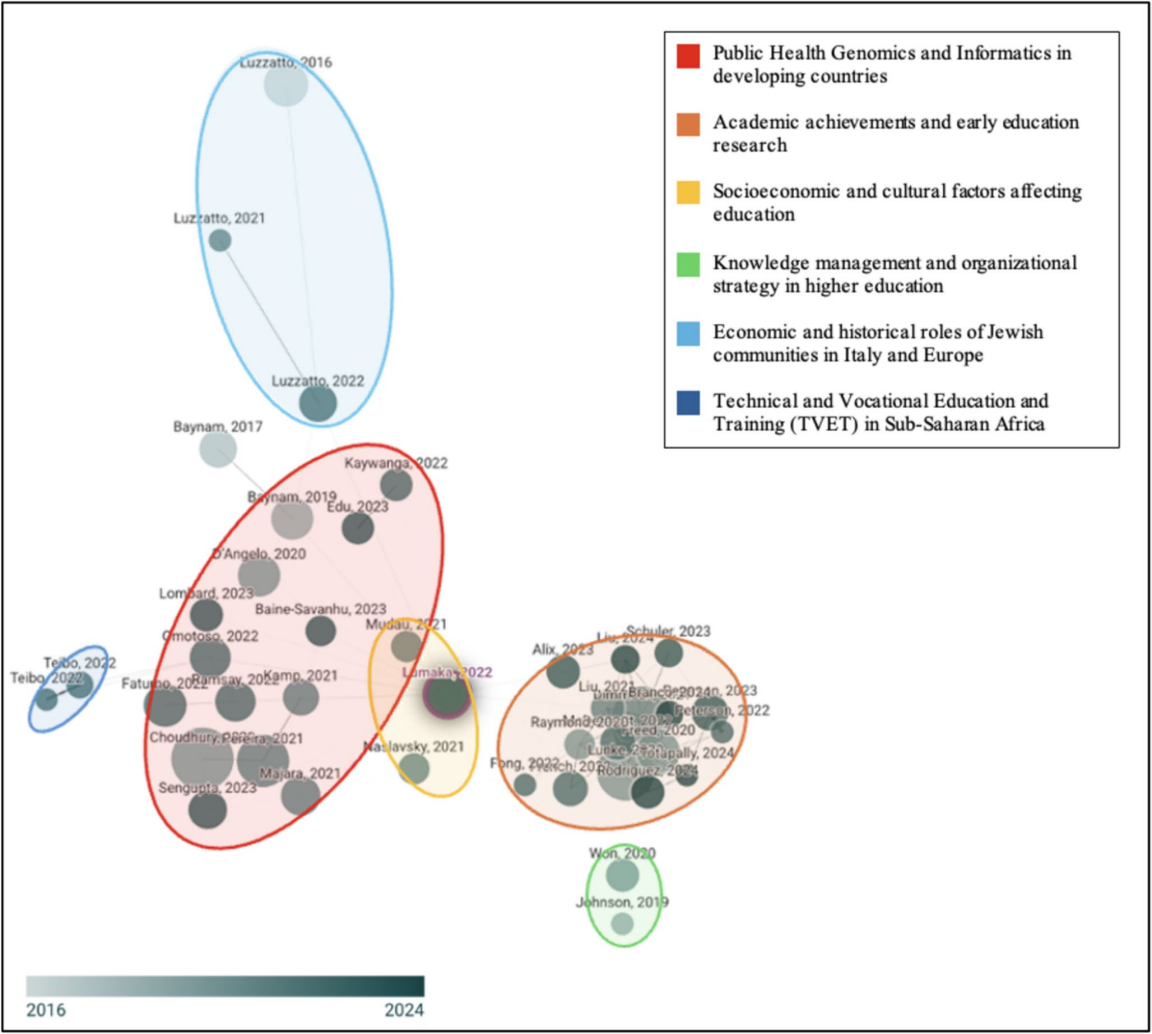
Distribution of publications between African-based and foreign-based researchers (Connect Papers, n.d.)

Upon finding articles from the PubMed database, a list of the resultant search was extracted, download and its input was exported and curated into a semi-structured spreadsheet in Microsoft Excel. Articles considered relevant to the research review topic, based on their title and the criteria listed in Table 1, remained on the list, while those that did not were eliminated. From the refined list of articles, their abstracts were screened to further evaluate if the content aligned with the subject points considered for discussion for the research review. Furthermore, a citation network analysis was conducted using a citation mapping tool called Connect Papers. The DOI of each included article was fed into the Connected Papers portal to develop the initial map, which was then clustered by region of research origin (Fig. 1). The use of this analysis tool established the relevance as well as the relationships amongst publications which enabled the identification of research clusters and thematic linkages within the field. Furthermore, this analysis tool highlighted two key observations: Firstly, that a significant portion of the literature is outdated and secondly, that there is a disproportionate number of articles that are published about the African populations from outside of the African continent.

### Inclusion and exclusion criteria for literature

**Table 1:** Shows the inclusion and exclusion criteria for an article to be considered for the research review

Inclusion criteria	Exclusion critria
English	Not in English
Open access	Not open access
Human participants were the main subjects	Used animals or plants as their main subjects
Qualitive studies that use focus group discussions interviews or other community engagement sources and/or critiqued research conditions	Qualitative studies without community engagement data or without empirical qualitative data (purely theoretical/methodological/opinion pieces)
Quantitative material that could be extracted to substantiate	No extractable qualitative data appropriate to substantiate
Published within the past 10 years (2015–2025)	Published before 2015
African context-focused	Not focused on African contexts. No analysis of African settings/populations

## Results

The following findings highlight the importance of culturally sensitive approaches in GGR particularly in the African contexts where traditional beliefs and social structures have a significant influence and shape community engagement and perceptions.

### Theme 1: Colonial legacies and the clash of medical paradigms

Understanding perceptions of GGR in African communities requires confronting the historical legacy of colonial medicine and the power imbalances that continue to infiltrate trust in biomedical research. The introduction of Western medicine into Africa is deeply tied to colonial power structures. Far from being a neutral intervention, it belied intentions to dominate both the bodily autonomy and traditional systems of indigenous knowledge of Africans. This colonial legacy continues to shape African attitudes and experiences towards healthcare today. For decades, the continent has long faced and grappled with poverty, under-resourced infrastructure and the persistent burden of diseases. Colonial powers not only succeeded in seizing Africa’s abundant resources but also exploited the very conditions they helped create, stripping communities of their humanity and bodily autonomy.

In the process, traditional healing practices were suppressed, paving the way for pseudo-scientific ideologies masqueraded as progress. These ideologies propelled oppressive systems such as colonialism, slavery, systematic racial oppression and eugenics. Ultimately, under the guise of medical research and clinical trials, African bodies were framed as different, even “disposable”, thus creating a false justification for exploitation (Tilley 2016). This narrative cemented a long-standing mistrust in biomedical research, especially considering how these medical experiments were often conducted in coercive and unethical ways on enslaved Africans (Braude 2009), directly contributing to the foundations of modern medical practices at an unbearable human cost.

Throughout the 20th and 21st centuries, the imposition of colonial health systems was exemplified by several unethical medical trials conducted across the continent, often dismissive of indigenous belief systems and societal norms (Viniegra-Velazquez 2020). Notable cases include South Africa’s Project Coast, carried out between the 1980 –1990 s during a period of institutionalised racial segregation, which involved the unethical testing of chemical and biological warfare agents on black civilians. In 1996, Pfizer’s Trovan Drug Trials in Nigeria were conducted on children without informed parental consent, resulting in disturbing fatalities and long-term disability effects. Similarly, controversial HIV/AIDS drug trials across the African continent during the early 2000s received backlash for the ethical violations and exploitative methodologies. Furthermore, several coerced sterilisation campaigns, such as those specifically targeting Namibian women during the early 1900s during German colonial rule. Then, finally, the research projects conducted on South Africa’s Khoi San communities exposed the ethical concerns regarding consent and bodily autonomy in scientific research (Schroeder et al. 2020). The histories underscore the violations of consent but deliberate oversight of Traditional African Medicine (TAM) practices, thus reinforcing a hierarchy privileging Western biomedical models and resulting in a power imbalance. As a result, several African communities remain reluctant and wary of modern research, particularly when it echoes familiar patterns of extraction without transparency.

Even after African nations gained independence, many healthcare systems remain built on European frameworks, perpetuating deep-rooted inequalities. Whilst modern research ethics has improved on paper, there is no dismissal as to how African communities’ participation was driven by blind deprivation. In highly technical fields such as Genetic and Genomic Research (GGR), many African communities continue to associate research with exploitation, therefore making participation difficult (Staunton and de Vries 2020).

Understanding the complex relationship of how power and science eclipse morality is essential. By confronting and readdressing these legacies and paradigms, we can begin to attend to and rebuild trust, redefine ethical standards and move towards research that intentionally serves the communities it claims to benefit.

### Theme 2: Underrepresentation of African populations in genomic research

Beyond historical mistrust, structural exclusion from global genomic research has further shaped how African populations experience and engage with GGR. Africa, the accepted birthplace of humankind is widely regarded as the epicentre of global genetic diversity (Street et al. 2008). Yet ironically, remains vastly underrepresented in GGR and biobanking efforts (Street et al. 2008). As of 2019, only 3% of genome data used for Genome-Wide Association Studies (GWAS) came from African populations – a figure that has drastically decreased to 1.1% as of 2021 (Omotoso et al. 2022).

Findings from our bibliographic study highlights the fragmented landscape between the local African researchers and their foreign counterparts (Fig. 1). While distinct clusters of African-based authors such as Mudau (2021) and Jimoh (2024) are present, the network also shows a significant number of publications from foreign researchers such as Liu (2023) and Schuler (2023). However, there is limited integration between these groups, which indicates a lack of cohesive collaboration. This fragmentation underscores the need for more inclusive and coordinated research efforts to effectively addresses the underrepresentation of African populations in genomic studies and to advance genomic equity across the African continent.

This gap in genomic sequencing capacity became particularly alarming during the COVID-19 pandemic. Africa contributed barely 2% of the total global sequence data, with 51% of it coming from just three countries: South Africa, Kenya and Nigeria (Omotoso et al. 2022). This inadequacy of diversity in global genomic databases has significant implications for the development and effectiveness of personalised medicine. Most genetic data used to develop diagnostics, and targeted treatments are derived from European ancestral groups. As a result, many therapies are often less effective or entirely unsuitable for African populations (Street et al. 2008). This excludes a significant portion of the global population from the benefits of precision medicine and ultimately reinforces existing health disparities.

**Fig. 2:**
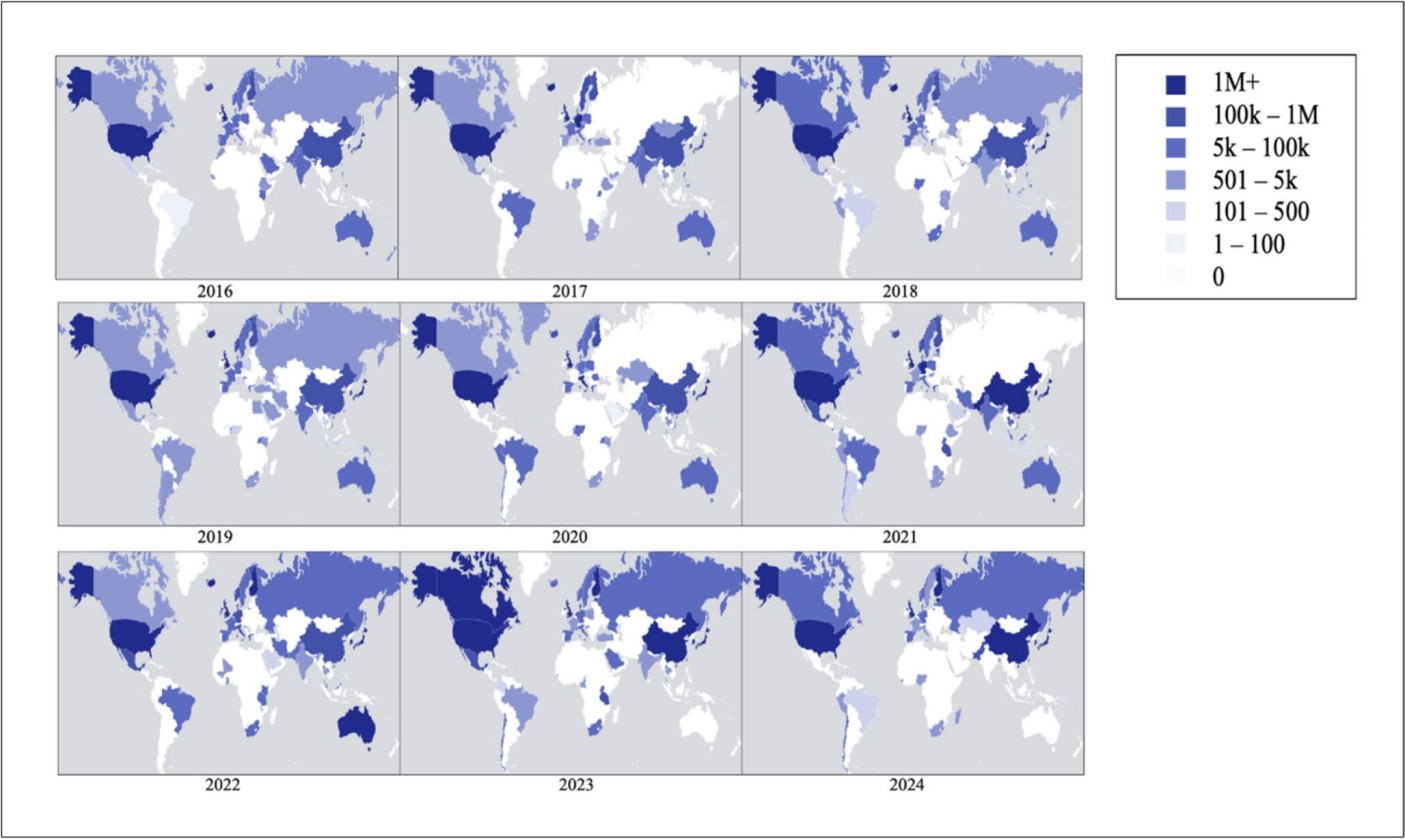
Geographical Distribution and Diversity of GWAS Participants, 2016–2024 (Mills and Rahal 2020)

A closer look at the GWAS data from 2016 to 2024, illustrated by Fig. 2, reveals the depth of this underrepresentation. World maps from the GWAS Diversity Monitor show a persistent and overwhelming concentration of participants in North America, Europe and parts of Asia, with African participation remaining minimal year after year. Only a handful of African countries appear in these datasets at all, underscoring the continent’s marginal inclusion in global genomic efforts.

**Fig. 3:**
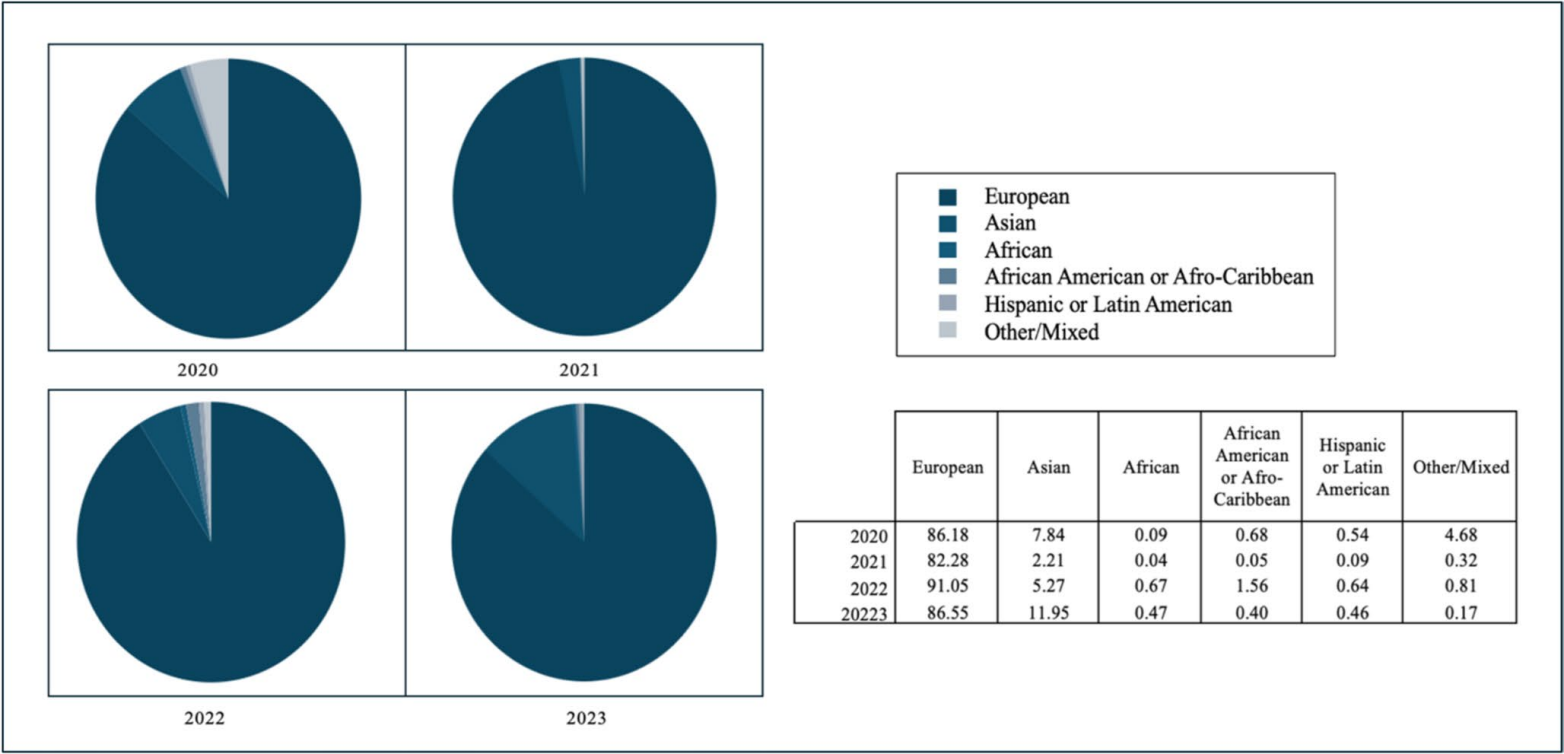
Ancestral Diversity of GWAS Participants, 2020–2023, (Mills and Rahal 2020)

Similarly, Fig. 3 demonstrates the ancestral imbalance. Between 2020 and 2023, individuals of European ancestry accounted for roughly 85% of all GWAS samples, while Asian participants averaged just under 9%. In contrast, African participants represented a mere 0.37%, African American/Afro-Caribbean individuals at 0.72%, Hispanic/Latin American at 0.51% and Other/Mixed ancestry at 1.46%. This profound skew towards European ancestry not only limits the applicability of genomic research findings but also risks exacerbating existing health inequalities if left unaddressed.

Improving diversity and representation in genomic databases is an ethical imperative. However, simply advocating for inclusion is not enough, and the efforts to diversify should be approached in a way that is culturally sensitive as well as historically aware. The hesitation of several African communities entrusting medical research is shaped not only by past exploitative practices but also by the deep-rooted sociocultural beliefs around health, illness and healing (Street et al. 2008).

As it has been established, purposeful Community engagement (CE) is a critical implication for building long-term trust and improving participation in GGR. From the onset of research projects, communities should be seen as partners rather than mere participants. Given the prevalence of misconceptions within local communities, it is essential to establish strong relationships with community leaders, as they play a key role in supporting the research process. Subsequently developing a semi-structured database with the aim of collecting statistical and contextual information from the community can enhance the effectiveness of the research project. This step is foundational in shaping participants’ attitudes towards research, especially considering that levels of engagement and willingness to participate often vary. Notably, individuals with some prior knowledge or awareness of local disease outbreaks are more likely to respond positively during the quantitative phases of research. This suggests that culturally grounded health literacy can significantly influence participation outcomes (Kripalani et al. 2019, 2021).

Table 2 below illustrates an example of the general demographic and contextual information that research teams typically collect during the quantitative phase of genomic research. These data categories are crucial for developing robust community engagement (CE) frameworks, which in turn improve participation rates. This approach is essential for aligning research design with the lived realities of local populations.

**Table 2:** Key Demographic and Contextual Data for Enhancing Community Engagement in Genomic Research

CATEGORY	EXAMPLE VARIABLES
Basic Demographic	Age, gender identity, nationality, ethnic group, language(s) spoken
Household Information	Household size, primary caregiver role, access to clean water
Health Literacy	Awareness of local disease, experience with healthcare services, vaccination history
Healthcare Access & Preferences	Primary source of healthcare (clinic/traditional healer/both/none), distance to healthcare facilities, frequency of care
Education & Work	Highest level of education, employment status, main source of income
Technological Access	Phone ownership, internet access, social media use, access to health apps
Cultural & Social	Religious identity, role of traditional healers, beliefs and perceptions about genetic research/illnesses
Personal Health Status	Disability status, chronic illness, history of medical mistrust

While this study incorporates exploratory citation network analysis to contextualise patterns of GGR in Africa, future works could further build on these findings through more robust bibliometric methods to systemically examine authorships, collaborations networks and publication trends in African genomics.

### Theme 3: Traditional medicine in Africa: relevance, challenges and opportunities

Perceptions of genomic medicine in African communities are also shaped by the enduring role of TAM, a practice which continues to inform how health, illness and healing are understood and practiced. Over 80% of Africans rely on TAM (Abbo et al. 2019) for their health needs, underscoring its enduring cultural and practical significance. This preference stems not only from accessibility but from the rooted cultural and spiritual resonance TAM offers – elements often absent in Western medical approaches. In many rural areas, illness is viewed not solely as a physical issue, but as a manifestation of spiritual or social imbalance, often linked to ancestors or other spiritual forces (Omonzejele 2008).

Traditional healers play a central role as primary caregivers, particularly in regions where Western biomedical services are geographically distant or financially inaccessible. Their guidance addresses not just the symptoms but the underlying causes of illnesses, often seen as spiritual or curses. Notably, TAM is a holistic system connected to culture, spirituality, community life and ancient wisdom (Abdullahi 2011). It aligns with how many Africans conceptualise health: as a state of balance between the spiritual, social and physical realms (Omonzejele 2008).

Knowledge of TAM is often passed down orally and experientially, through storytelling, ritual and ancestral guidance (Rehfuess et al. 2019). Elders play a crucial role in this transmission, preserving cultural identity and ensuring the continuity of healing traditions (Mzimela and Moyo 2024). While such knowledge may not conform to the Western academic paradigms, dismissing traditional practitioners as “illiterate” or unscientific overlooks the legitimacy and empirical richness of their practices.

TAM continues to serve as a primary or only form of healthcare for millions across the continent. In regions such as Limpopo, Eastern Cape, and Northern Ghana, where biomedical infrastructure is sparse. Remedies are typically locally sourced, inexpensive and tailored to specific community needs (Ahmed et al. 2023). A wide array of wild plants including *Rooibos*, *Hypoxis hemerocallidea*, and *Sutherlandia frutescens* are traded informally in South African markets (Street et al. 2008). Preparation of these remedies often follows ritualistic protocols, from harvesting at specific times to ceremonial cleaning and ancestral guidance (Street et al. 2008). These rituals shape the user’s belief in the remedy’s healing power, deeply affecting mental and emotional well-being.

However, TAM faces challenges of its own. Concerns about the lack of standardisation, quality control, and potential risks associated with imprecise dosages or unhygienic preparation practices (Abdullahi 2011) remain prevalent. Furthermore, ongoing tensions between traditional and Western medicine in Africa have long existed, fuelled by cultural misunderstanding. Because TAM knowledge is primarily oral and informally transmitted (Rehfuess et al. 2019), formal documentation remains limited. Coupled with decades of colonial suppression and marginalisation, this has led to a shortage of formally trained and licensed traditional practitioners.

Integrating spirituality into Western medical frameworks also presents cultural and philosophical barriers. Many African communities’ reliance on TAM stems from a complex mix of cultural identity, financial constraints and geographical limitations (Omonzejele 2008). For researchers and policymakers, understanding these dynamics, particularly the spiritual connections, is essential for developing healthcare systems that are ethically sound and culturally responsive to the needs of African communities. Respecting traditional practices requires intentional and open dialogue between healthcare systems (Collaborators 2024).

Despite these challenges, many patients already engage with both healthcare systems, illustrating a neutral openness to blending the two healthcare approaches. The willingness presents an opportunity for collaboration. Organisations like the World Health Organisation (WHO) advocate for the integration of traditional medicine into national health policies. Some medical schools now recognise and explore ways to incorporate indigenous knowledge into curricula, fostering cultural competency and improving patient-provider relationships (Ikhoyameh et al. 2024).

With collaboration, mutual respect and cultural humility between conventional and traditional healthcare systems, we can forge a complementary model of care that better serves diverse African populations.

### Theme 4: The weigth of stigma and discussion around genetic predisposition

Cultural interpretations of inheritable predispositions contribute significantly to stigma, which in turn influences how individuals and families respond to genetic information, often resulting in social consequences such as marginalisation or isolation. In many African communities, the idea of genetic predispositions is often entangled with spiritual, social or moral beliefs. Illness may be labelled as a reflection of ancestral punishment, spiritual imbalance or a form of “bad blood” passed down generations (Moore 2024). These narratives carry deep social weight, affecting family reputation, self-worth and standing in the community.

For instance, conditions such as mental illnesses, which may have a genetic basis, are sometimes attributed to witchcraft, leaving individuals feeling fearful and isolated rather than seeking support. (Shange and Ross 2022). In South Africa, GGR participants have expressed their concern of being “marked” by a genetic predisposition, wary of stigmatisation for both them and their entire families. This reflects how diagnoses carry a heavier weight beyond their medical implications (Wessels et al. 2024). Such stigma makes open dialogue about predisposition risks difficult. Resulting in the many cases in which individuals may hesitate to consult health professionals, participate in research or even share their diagnoses. In African contexts, the question of *who* should be informed of genetic results brings further ethical complexity, especially how it may affect marriageability, privacy or community standing (Suter 2020). It rarely affects just the individual; it impacts the entire family system.

In cultures where marriage is both a rite of passage and a form of social capital, the risk or presence of a genetic predisposition could severely ruin one’s eligibility. Reinforcing silence and secrecy to avoid being branded as “tainted” or having “bad blood” (Sikhwari and Mudau 2024). This apprehension may cause individuals to hide genetic information, even from close relatives or partners, creating tension between personal privacy and social harmony.

Moreover, these tensions are compounded by past breaches of trust, such as data mismanagement or sample reuse, which have reinforced anxieties around sovereignty and misuse. In response, South Africa’s ethical guidelines stress that GGR must never contribute to or worsen existing stigma (Amayoa et al. 2022). This is particularly crucial in African settings where health information often reverberates beyond the individual, affecting the family and wider community. Western ethics, which prioritize individual autonomy, may can clash with African values that advocate for collective decision-making. This then creates a uniquely complex ethical terrain for both genetic research and counselling across the continent.

### Theme 5: Structural and economic constraints in access to genomic research

Both physical and systemic barriers significantly limit participation in GGR and access to genetic services across many parts of Africa. Many Africans live far from specialised healthcare services, especially those related to genetics, which are mostly concentrated in urban centres (Wigley et al. 2020). This geographic isolation creates a substantial travel burden, with hidden costs such as accommodation, childcare and most critically, time away from work. These factors make it difficult for people in remote areas to access necessary care.

Additionally, inadequate medical infrastructure – including under-equipped laboratories, limited specialised clinics and unreliable electricity poses a major challenge in conducting complex genomic research or delivering advanced genetic testing and counselling (Fatumo et al. 2022). Many healthcare facilities also lack stable internet connectivity, which further hinders service delivery. These infrastructural gaps are compounded by the shortage of education and training among healthcare professionals, many of whom are not adequately prepared to understand or apply GGR in clinical settings (Tindana et al. 2017b). This knowledge gap can fuel distrust, misconceptions and reluctance within communities to engage in research or make use of genetic services, ultimately reinforcing a cycle of exclusion and stalling medical progress (Wonkam et al. 2006).

As a result, access to genetic services and counselling is severely limited, particularly for individuals residing in rural regions. In many African countries, such services remain scarce and often restricted to urban centres, leaving rural communities underserved or entirely excluded (Mulder 2017). For instance, some countries may only have a handful of trained genetic counsellors, typically located in major cities. One of the main contributors to this shortage is ‘brain drain’ – the migration of skilled professionals to other regions or countries overseas in search of better opportunities, leaving local healthcare systems understaffed (Omotoso et al. 2022). Consequently, this leaves many individuals with genetic risks or conditions undiagnosed, untreated or unaware of vital information that could improve their health outcomes.

Another major barrier to the advancement of genomic medicine in Africa is the lack of investment and engagement from the pharmaceutical and biotechnology industries. Large pharmaceutical companies often prioritise high-income nations because of their higher commercial value. Despite Africa’s remarkable genetic diversity, only 3.3% of global clinical trials are conducted on the continent (Fatumo et al. 2022). Without the support from corporate investment, it not only limits the development of therapies tailored to African populations but also undermines the inclusion of other genetically diverse sub-populations worldwide. Therefore, this exclusion not only reinforces historical injustices but also compromises global health equity.

Addressing these issues and challenges requires intentional and sustained investment in African-led research, stronger pharmaceutical policies and meaningful incentives to promote the development of treatments that are relevant to Africa’s health needs. Initiatives such as H3Africa aim to improve local research capacity, expand genomic data collection and train healthcare professionals in genomics. However, such efforts need consistent support and must be accompanied by broader policy reforms (Mulder 2017).

The inequalities in access to healthcare and genetic services underscore broader health disparities across the continent, reinforcing the perception that advanced care is a privilege reserved for urban elites rather than a fundamental right. Without deliberate efforts to extend services into rural communities and strengthen genomic literacy at the local level, the benefits of genomic medicine will remain inaccessible to reach to most Africans. Transforming these systematic and economic barriers into opportunities for equity requires long-term investment in local healthcare systems and locally grounded service models that are thoughtfully aligned with – and meaningfully integrated into – local cultural values.

### Implications for research and practice

Building on to the discussed findings of this review, there are several implications for research and practice to be considered. These following implications emphasise the need for contextually grounded, culturally responsive, and ethically robust approaches to GGR, particularly in readdressing historical inequities, fostering long-term trust and improving equitable access across communities.

### Cultural sensitivity as a foundation for trust in genomic research

To reduce wariness and misconceptions about genetic risks, educational efforts must be culturally grounded, inclusive and accessible. Creating safe spaces for open dialogue within African communities is key, serving as a powerful bridge to trust. Community leaders play a crucial role in raising awareness and explaining the complexities of science in ways that feel familiar and understandable (Nankya et al. 2024).

Researchers and stakeholders must go beyond simply presenting facts. They need to listen and actively engage with the communities’ values and concerns. Culturally sensitive education involves being able to break down these complex genetic concepts into relatable, jargon-free language, while also reframing harmful beliefs, such as viewing genetic conditions as “bad blood” or “curses.” Community-based initiatives are essential for shifting perspectives, deepening long-term understanding and overcoming language and cultural barriers (Oladayo et al. 2024). In South Africa, genetic counsellors play a pivotal role, not only in educating the public about genetic conditions but also in providing emotional support and connecting individuals to relevant support networks (Nankya et al. 2024). These efforts ensure that accurate information is shared in ways that resonate with the community.

Creating that safe space where people feel comfortable discussing genetic health without fear or stigma promotes dignity and empowers proactive health management. Equitable access to genetic counselling should not be seen as a luxury, but as a necessity for inclusive, stigma-free health care. Ultimately, clear communication and cultural sensitivity are foundational pillars for building trust, enabling fair participation in GGR and ensuring that advancements benefit African communities without undermining their cultural dignity.

Tackling these systematic shortcomings demand a holistic strategy rooted in education, local empowerment and continuity of care. Without consistent follow-ups, early efforts risk becoming extractive or transactional, undermining trust and cutting off the very benefits research promises to deliver (Mubarak and Ashraf 2024). Therefore, training local practitioners such as community health workers and nurses alongside traditional healers in basic genomic literacy and ethical communication, is crucial. When communities see their own members providing information in culturally relevant ways, they are more likely to engage meaningfully and benefit fully from such initiatives.

### Community engagement and participatory research

For genomic research to truly improve health outcomes it depends heavily on genuine community involvement and participatory approaches. By adopting cultural sensitivity and humility and recognising the local customs, GGR can meaningfully serve African communities (Matshabane et al. 2022). Cultural humility involves ongoing self-reflection, openness to learning from others and recognising one’s own biases. In several African cultures, personal identity is deeply rooted within the broad context of family, community, and spirituality. The powerful Xhosa proverb, “Umntu ngumntu ngabantu” – I am, because we are – reflects the worldview rooted in interconnectedness, shared humanity and collective well-being (Matshabane et al. 2022). This ethos promotes inclusivity in research practices, dismantles historical power imbalances in research and builds more honest partnerships (Matshabane et al. 2022).

Community-Based Participatory Research (CBPR) is a collaborative model in which researchers and community members work together as equal partners to address shared health challenges. This approach empowers communities and leads to health solutions that reflect local values and realities (Kamanda et al. 2013). In South Africa for example, the “Drama of DNA” was an initiative that used theatre to explore the ethical complexities of returning genetic research results, tailoring performances for local audiences (Faure et al. 2020). Moreover, the San people of Southern Africa, a population that has long been involved in genetic research, developed their own code of ethics which just demonstrates a powerful example of community agency and taking ownership in research.

Equitable benefit-sharing is a core principle in genomic research, ensuring that positive outcomes – whether in the form of sharing of intellectual property, technology transfer or infrastructure development – are distributed. These benefits must reach local scientists, research participants and their communities alike, preventing the exploitation of human biological samples as mere commodities for external gain (Omotoso et al. 2022). The long-term engagement, mutual respect and transparent collaboration offer powerful tools for reshaping health research across Africa into a more community-driven and inclusive process.

Strong community relationships break down cultural and social barriers and improve communication between healthcare providers and community members (Olakunle Saheed et al. 2025). When researchers involve communities from the very beginning, they gain invaluable insights into what the public actually needs and preferences, which then helps tailor services and research questions to address specific health challenges (Bategereza et al. 2021). This collaborative approach creates shared ownership, integrates local knowledge and leads to making more effective interventions. But to build this trust, “once-off projects” are not enough. Ongoing partnerships are essential for addressing past mistrust and creating lasting, equitable health outcomes.

However, securing informed consent that is both ethical and culturally appropriate remains a challenge. The complexity of genomic research, along with poverty, varying literacy levels, and vast cultural-linguistic diversity, makes it difficult for participants to fully grasp what they’re consenting to (Munung et al. 2016). Many struggle to understand concepts such as how biological samples will be stored and used in the future, potential risks that may rise, including stigma or misuse of data. While participants may think they have understood what they’re consenting to, research rather shows that participants often miss the key concepts, especially pertaining to data use, privacy and long-term risks (Amayoa et al. 2022). Translating scientific language into local dialects poses another challenge, which often leads to distortion or loss of its intended meaning of “Broad consent,” where participants agree to future unspecified use of samples, is becoming more common. However, it must be handled responsibly with clear information, strong oversight and ongoing dialogue with communities (de Vries et al. 2016).

**Fig. 4:**
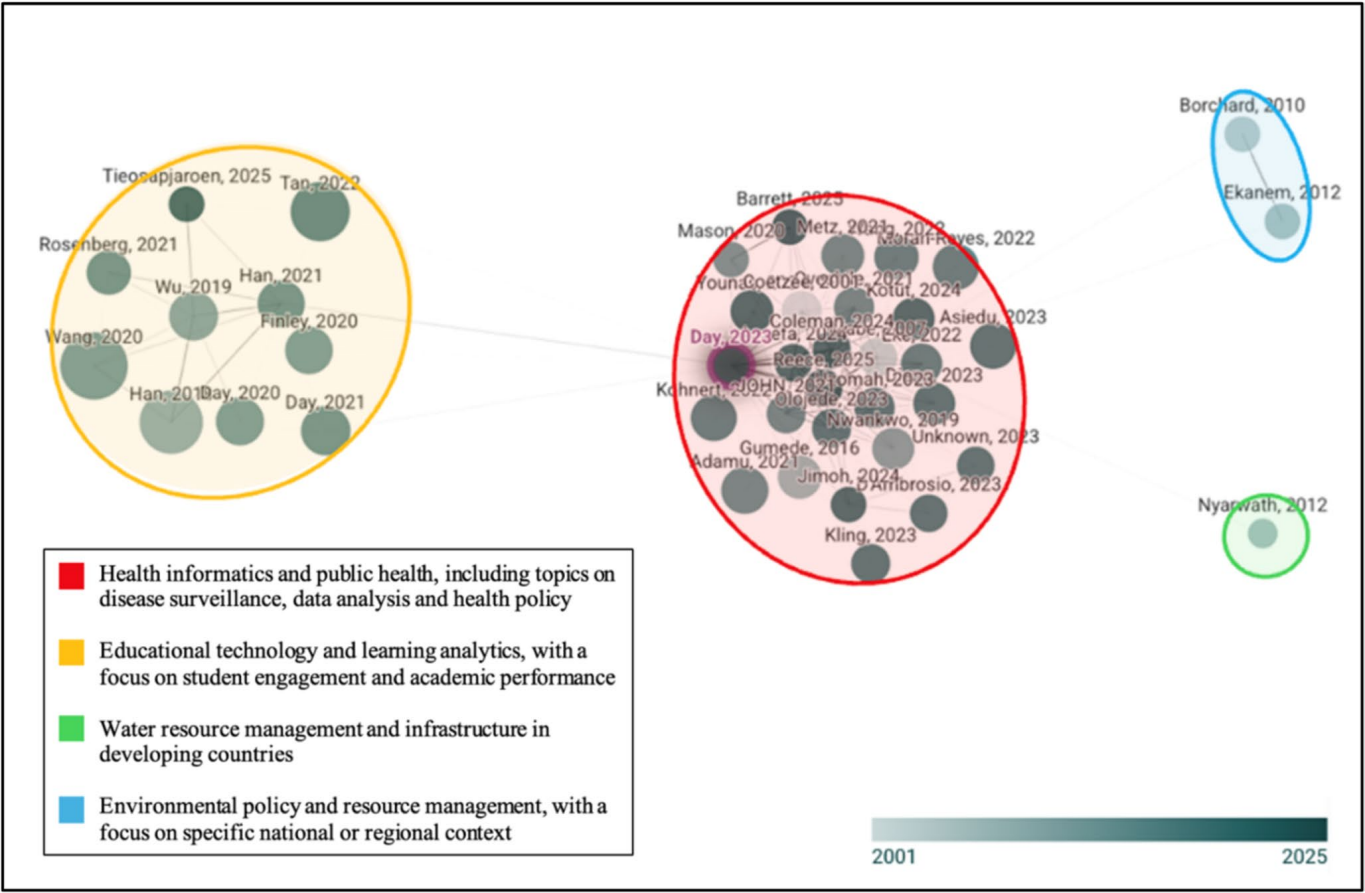
The Intellectual structure of Community Engagement and Participatory Research (CEPR) literature in African contexts (Connect Papers, n.d.)

For this review, we conducted a second citation network analysis that illustrates the intellectual structure of the literature on Community Engagement and Participatory Research (CEPR) in African contexts (Fig. 4). At the centre is a densely interconnected central cluster of recent publications such as Day (2023), Kohnert (2020) and Barrett (2025), which actively build and link with one another. In contrast, peripheral clusters anchored by earlier works, including Borchard (2010) and Ekanem (2012), show more nuanced subfields that initially shaped the discourse. The colour gradient of the nodes – ranging from lighter shades for older publications to darker ones for recent publications – visually tracks the evolution of the field. Node size, which corresponds to citation count, highlights the studies that have had a significant influence on the research agenda. Holistically, the visualisation reflects the shift towards inclusivity in the field. The emergence of African-led signals a growing foundation for participatory models that are rooted in community trust, contextual relevance and shared decision-making.

Genuine engagement is a multi-step process that goes far beyond simply informing or consulting communities. It requires active collaboration, shared decision-making and building trust over time. Researchers must show cultural sensitivity, communicate openly and consistently share findings with communities. Organisations emphasise a shift in healthcare culture, one that prioritises knowledge co-creation over extractive research practices (Sheldon et al. 2024). This means offering flexible support, designing culturally relevant studies and creating space for continuous feedback and dialogue (Tembo et al. 2021). By promoting two-way communication, building research capacity within communities and involving a diverse range of local stakeholders, researchers can establish the trust needed for truly ethical and impactful genomic health initiatives in Africa.

### Equitable genomics through ethical governance and regulatory reform

To ensure that GGR benefits African populations requires robust ethical governance and regulatory frameworks that protect community interests and promote accountability. The H3Africa Ethics and Governance Framework for Best Practice in Genomic Research and Biobanking in Africa addressed how to consider both financial and non-financial benefits in the context of genomic research and biobanking (Consortium et al. 2014). It emphasises aligning these benefits with African cultural values and community expectations. Given the continent’s vast genetic diversity, increasing African representation in genomic databases is not only a matter of fairness but also critical to advancing global health equity (Ridgeway et al. 2019).

A key ethical concern in genomic research is ensuring that communities maintain control over their biological samples and associated data. While genetic data from African populations holds immense scientific value, its management must be carefully regulated to prevent misuse (Scott et al. 2020). Committees such as H3Africa Data and Biospecimen Access Committee (H3A-DBAC) play a pivotal role in this role by reviewing data access requests to ensure responsible use, alignment with local laws and respect for the self-governance of source countries (Scott et al. 2020). Other initiatives such as eLwazis, an African-led open data science platform, similarly promote local data storage and analysis. Thereby, supporting regional innovation and long-term sustainability (Sibomana 2024).

Although ethical guidelines – such as broad consent, data sharing and community engagement – have been established by H3Africa consortium, gaps remain (Staunton et al. 2019). As acknowledged in Sect.  4.2 and 6 of the H3Africa Ethics and Governance Framework, there is a lack of robust national legislation to govern GGR and biobanking effectively across several African countries (Ethics 2017). In several instances, regulatory structures are outdated, non-existent or vague (Tindana, Campbell, Marshall, Littler, Vincent, Seeley, de Vries, et al., 2017). Many existing cases were drafted before the emergence of large-scale genomics projects and biobanking, leaving has resulted in leaving critical grey areas in data governance, ownership and cross-border data sharing (Consortium et al. 2014). These legal loopholes give way for the export of biological samples and data with minimal oversight. This further raise concerns around exploitation, inequity and the erosion of community autonomy.

Moreover, even where national regulations exist, they are often inconsistently enforced or linguistically inaccessible. The capacity of national ethics committees across Africa varies widely, with some lacking the technical infrastructure or expertise to oversee genomic studies effectively (Consortium et al. 2014; Ethics 2017) Furthermore, this fragmentation is further exacerbated by the limited availability of scientific and legal terminology in the indigenous languages, thus making it difficult for communities to fully understand the scope and implications of research participation. In contrast, these regulatory inconsistencies make it easier for researchers to bypass community-centred safeguards, undermining ethical protections and complicating cross-border collaborations (Consortium et al. 2014). Without enforceable national laws, even the most comprehensive ethical guidelines risk being as aspirational rather than actionable.

Another pressing concern is the lack of legal enforcement mechanisms for benefit-sharing. Although ethical frameworks advocate for equitable sharing of research outcomes and benefits, Sect.  4.3 of the H3Africa Ethics and Governance Framework make it clear that these provisions are often not legally binding, and as a result, few legal instruments require researchers to share the advantages or outcomes of their research findings. This means benefit-sharing – whether through co-authorship, infrastructure support or skill development – remains largely at the discretion of researchers and sponsors (Consortium et al. 2014). The absence of enforceable obligations risks perpetuating systematic inequalities, excluding African communities from the long-term scientific and socio-economic benefits, and ultimately reinforcing the very mistrust that many sovereignties seek to dismantle.

Addressing these challenges requires both national-level reforms. At the national level, ethical frameworks must be updated to regulate genomic research and data governance effectively. Simultaneously, efforts to establish a coordinated Pan-African regulatory framework are essential to safeguard autonomy, ensure equitable benefit-sharing and prevent regulatory loopholes that compromise ethical standards.

In moving from principle to practice, researchers and stakeholders can take several steps to uphold these ethical commitments. First, meaningful engagement with local leaders and traditional healers is vital, as they serve as trusted gatekeepers, provided that their involvement and insights ensure that research respects community values and traditions (Bukini et al. 2019). Second, researchers must acknowledge and incorporate traditional health knowledge, notably a primary healthcare source in several communities, into research and health education. This integration can strengthen community trust and enhance outcomes (Street et al. 2008). Lastly, co-designing research studies with community members ensures projects reflect local needs, values and priorities (Khan et al. 2024). While this approach demands time and requires open dialogue, it remains fundamental to producing ethical and impactful research. By embedding cultural humility, ethical accountability and authentic collaboration into every phase of research, African researchers and stakeholders have the potential to lead the way towards a transformative and inclusive genomic future (Mackworth-Young et al. 2022).

## Conclusion

The integration of genomic medicine into African healthcare systems is multifaceted and thus requires a culturally informed and historically aware approach. Readdressing stigma, complexities of disclosure and accessibility must go in tandem with dismantling systematic barriers – especially in rural and underserved communities. Without this, equity cannot be achieved without intentionally attending to these structural gaps. Central to this effort is the establishment of authentic community partnership. Community members should be considered as co-creators in research design, valued for their indigenous knowledge, lived experience, data governance and benefits-sharing. Frameworks established by initiatives like H3Africa and values such as Ubuntu offer ethical pathways that honour Africa’s dignity, diversity and voice. By ensuring science serves communities rather than merely producing outcomes, the potential of genomic medicine and research in Africa can evolve beyond being a frontier of innovation. The very cradle of humankind has the potential not only to lead scientific progress but also to transform lives, ethically and equitably.

## Data Availability

All data is available upon reasonable request. The data generated herein is bibliographical in nature.
